# Phenotyping the Snark: hazards of 3D

**DOI:** 10.1186/s12915-016-0324-8

**Published:** 2016-11-18

**Authors:** Graham Bell

**Affiliations:** BMC Biology, London, UK

## Background

Figures in articles should fairly represent the underlying data, but different types of chart used to present the information can work less well than others. Pie charts can be particularly hard to interpret; and 3D charts, whilst visually striking, generally serve only to obscure the message from the data. This article provides examples of both problems.

## Commentary

In a follow-up to their highly cited paper presenting a reliable method to accurately distinguish the much-hunted Snark from the closely related but toxic Boojum [1], Professors Lewis and Carroll sought to describe the behaviour of the elusive and hitherto unstudied Snark.

**Fig. 1.**
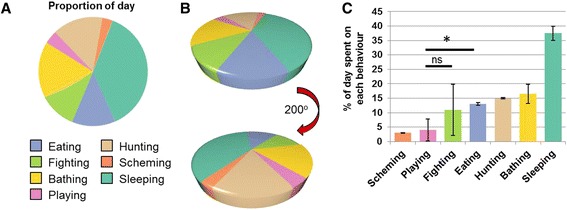
The typical time spent by a Snark engaging in its characteristic activities as a proportion of one day. **a** Data represented as a 2D pie chart. **b** Data represented as a 3D pie chart, in the same orientation as **a** (*top*) and the same data rotated 200 degrees (*bottom*). **c** The same data plotted as a bar char. *Error bars* represent the standard deviation of three independent experiments. *P < 0.05, Student’s *t*-test; *ns* not significant

Figure 1 shows the proportion of a typical 24-h period spent engaging in characteristic activities, which the authors represented in a pie chart (Fig. 1a). However, pie charts can make it difficult to interpret the results if the numerical data (percentages) are not included. In this example, it is hard to tell whether more time was spent eating (blue) or fighting (green); or more time playing (pink) or scheming (orange).

Data plotted in a 3D pie chart are even harder to interpret because the perspective can make segments appear different sizes. In Fig. 1b it seems that more time is spent eating (blue) than hunting (brown) or bathing (yellow). However, simply rotating the chart makes the data appear very different, with hunting now seemingly a significantly larger fraction of overall time than eating or bathing.

A bar chart can be a simpler way to compare values (Fig. 1c). Now one can easily see the differences and, in contrast to the 3D chart, that more time is spent bathing than either hunting or eating.

It is also less easy to show variation within data when represented as a pie chart. In Fig. 1, the pie chart shows that there is a large difference in the time spent fighting (green) and the time spent playing (pink). However, the bar chart includes error bars and *p* values, based on the three independent experiments that the authors performed, indicating that there is substantial variation in the data for both behaviours and that there is no statistically significant difference between the means. Of course, bar charts, *p* values and the interpretation of “statistically significant” have their own problems, as discussed in previous pieces in this series [2, 3], but pie charts are particularly prone to obscuring the issues.

**Fig. 2.**
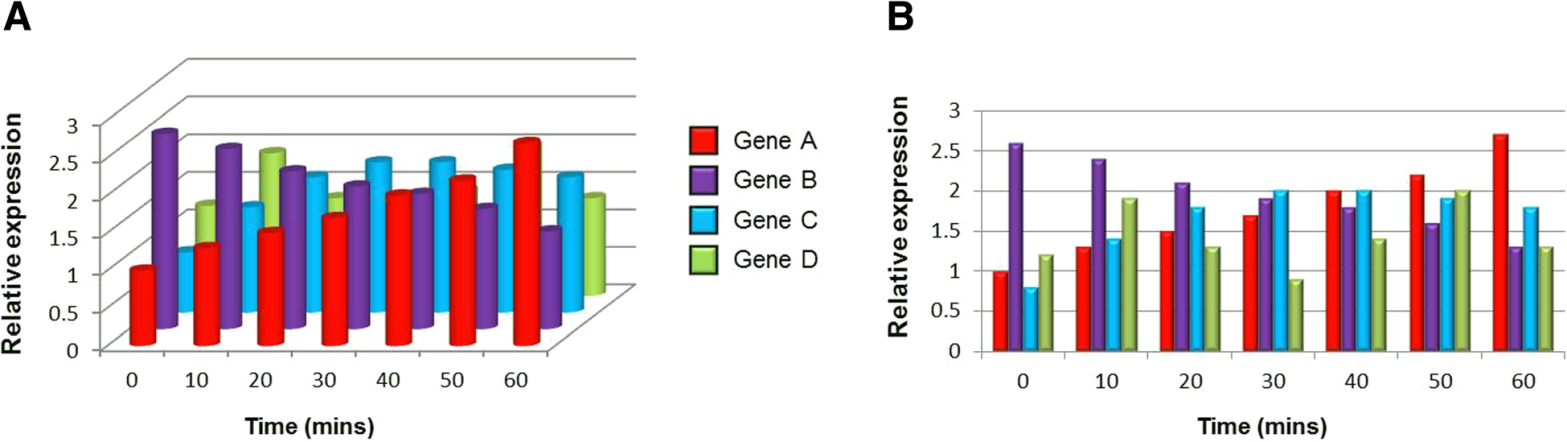
Relative expression levels of four genes across a 60-minute time course experiment in a developing Snark embryo. The same data are plotted as a 3D (**a**) or 2D (**b**) bar chart

Just as a pie chart can be made more confusing represented in 3D, so too can bar charts lose clarity, despite—arguably—appearing more aesthetically pleasing. Fig. 2 shows the expression of four genes in a developing Snark embryo in a time course experiment, represented as 3D (Fig. 2a) or 2D (Fig. 2b) data.

In the 3D chart, it is hard to see what values the bars represent. At time 0, gene A clearly looks like a value of less than 1; however, the same data plotted in a 2D chart shows that gene A at time 0 is in fact 1. At T = 40 minutes, the 3D chart seems to show similar expression of genes A and B, both less than gene C: the 2D chart reveals that it is genes A and C that are expressed at the same level, and both higher than gene B. Lastly, the levels of gene D (green) are often impossible to see in the 3D chart, being hidden behind the other bars.
